# EMPATHY MODERATES THE RELATIONSHIP BETWEEN RECIPROCITY AND STRESS AMONG AGING PARENTS AND CHILDREN

**DOI:** 10.1093/geroni/igae098.1602

**Published:** 2024-12-31

**Authors:** Da Jiang, Zewen Huang, Xinyuan Peng

**Affiliations:** The Education University of Hong Kong, Hong Kong, China (People’s Republic); The Education University of Hong Kong, Hong Kong, China (People’s Republic); The Education University of Hong Kong, Hong Kong, China (People’s Republic)

## Abstract

Previous studies examining the relationship between being under-benefitted and stress among aging parents and their adult children have yielded mixed findings. Few studies have examined whether this positive association can be alleviated by state-level or trait-level factors. Given the positive effects of empathy on interpersonal exchanges, we tested the moderating role of empathy on the relationship between being under-benefitted and perceived stress among aging parents and adult children in this 14-day diary study. A sample of 99 pairs of parents (Mage=50.01, SDage=4.53; 79.8% female) and children (Mage=22.38, SDage=3.49; 85.9% female) were recruited to report their level of being under-benefited in the exchange with their parent/child, perceived stress, and empathy as an affective state on a daily basis for 14 consecutive days, after completing a pretest which measured their trait empathy and demographic information. For both parents and children, the positive under-benefited-stress association was only significant when they reported lower empathy as a daily affect. The under-benefitted-stress link was negative when children reported greater daily empathy. Children reported more perceived stress on the days their parents reported a greater level of being under-benefitted. Such association was only significant in children with lower trait empathy. The negative association between children’s being under-benefitted and parents’ perceived stress was only significant in parents with higher trait empathy. These findings highlight the importance of empathy as a daily affect and a trait in the relationship between daily exchanges and mental health in the intergenerational contexts between aging parents and adult children.

